# Pan-Cancer Analysis Reveals Alternative Splicing Characteristics Associated With Immune-Related Adverse Events Elicited by Checkpoint Immunotherapy

**DOI:** 10.3389/fphar.2021.797852

**Published:** 2021-11-24

**Authors:** Xiujing He, Jing Yu, Hubing Shi

**Affiliations:** Laboratory of Tumor Targeted and Immune Therapy, Clinical Research Center for Breast, State Key Laboratory of Biotherapy, West China Hospital, Sichuan University and Collaborative Innovation Center, Chengdu, China

**Keywords:** immune-related adverse events, immune checkpoint inhibitors, cancer immunotherapy, alternative splicing, splicing isoforms

## Abstract

Immune-related adverse events (irAEs) can impair the effectiveness and safety of immune checkpoint inhibitors (ICIs) and restrict the clinical applications of ICIs in oncology. The predictive biomarkers of irAE are urgently required for early diagnosis and subsequent management. The exact mechanism underlying irAEs remains to be fully elucidated, and the availability of predictive biomarkers is limited. Herein, we performed data mining by combining pharmacovigilance data and pan-cancer transcriptomic information to illustrate the relationships between alternative splicing characteristics and irAE risk of ICIs. Four distinct classes of splicing characteristics considered were associated with splicing factors, neoantigens, splicing isoforms, and splicing levels. Correlation analysis confirmed that expression levels of splicing factors were predictive of irAE risk. Adding *DHX16* expression to the bivariate PD-L1 protein expression-fPD1 model markedly enhanced the prediction for irAE. Furthermore, we identified 668 and 1,131 potential predictors based on the correlation of the incidence of irAEs with splicing frequency and isoform expression, respectively. The functional analysis revealed that alternative splicing might contribute to irAE pathogenesis via coordinating innate and adaptive immunity. Remarkably, autoimmune-related genes and autoantigens were preferentially over-represented in these predictors for irAE, suggesting a close link between autoimmunity and irAE occurrence. In addition, we established a trivariate model composed of CDC42EP3-206, TMEM138-211, and IRX3-202, that could better predict the risk of irAE across various cancer types, indicating a potential application as promising biomarkers for irAE. Our study not only highlights the clinical relevance of alternative splicing for irAE development during checkpoint immunotherapy but also sheds new light on the mechanisms underlying irAEs.

## Introduction

Immune checkpoint inhibitors (ICIs) therapy has revolutionized the therapeutic landscape in oncology. These inhibitory drugs targeting immune checkpoints, such as cytotoxic T-lymphocyte-associated protein 4 (CTLA-4), programmed cell death protein 1 (PD-1), and its ligand (PD-L1), can bring long-lasting clinical benefits by eliciting immune responses against tumors in the treatment of various cancer entities (Bagchi et al., 2021). However, their application has been curtailed by incredibly diverse immune-related adverse events (irAEs) in the clinic. IrAEs can potentially affect almost every organ system and lead to fatal consequences in some cases (Postow et al., 2018). Most commonly, these fatal effects clinically manifest as colitis, hepatitis, pneumonitis, myocarditis, and neurologic effects (Wang et al., 2018). Among them, colitis is highly predominant and accounts for 70% of anti-CTLA-4-related fatalities, whereas anti-PD-1/PD-L1-related fatalities are often from pneumonitis, which results in 35% mortality (Wang et al., 2018). Therefore, early detection and diagnosis of irAEs as well as aggressive management are urgently required.

To improve patient selection and safety for ICI applications, numerous efforts to discover candidate biomarkers for irAE diagnosis and prediction are currently ongoing, which are essential to tailor safety monitoring protocols and treatment decisions. Nonetheless, studies on the mechanism of irAE onset and the corresponding biomarker development are still in the exploratory stage. Fortunately, several possible biomarkers for irAEs have been suggested, such as T-cell receptor (TCR) diversity (Johnson et al., 2016), CD8 T-cell clonal expansion (Subudhi et al., 2016), and tumor mutational burden (TMB) (Bomze et al., 2019). However, none of these factors is sufficient to achieve accurate risk prediction of irAEs and predictive performance needs to be evaluated in larger patient cohorts with ICI regimens. Hence, the identification of reliable biomarkers for irAEs prediction remains a critical challenge in cancer immunotherapy.

The FDA adverse event report system (FAERS) database, allowing relatively unbiased estimates for the relative risk for specific adverse events based on real-life patient populations, has been increasingly applied to biomarker discovery for drug adverse events (Bomze et al., 2019; Jing et al., 2020; Kerepesi et al., 2020; Van Hasselt et al., 2020). An elegant study revealed the significant association between TMB and irAEs occurrence during PD-1 immunotherapy by integrating real-world pharmacovigilance data from the FAERS database and mutation feature (Bomze et al., 2019). Similar strategies have also been successfully used to identify the additional biomarkers for irAEs (Bomze et al., 2019; Jing et al., 2020; Kerepesi et al., 2020), suggesting this strategy is robust and efficient, especially in the absence of a patient-sample cohort with sufficient sample size. The predictive potential of transcriptomic changes for irAEs has been demonstrated using this strategy, in which lymphocyte cytosolic protein 1 (*LCP1*) and adenosine diphosphate dependent glucokinase (*ADPGK*) served as biomarkers for irAE prediction by evaluating the correlation between multi-omics factors and irAE reporting odds ratios (ROR) calculated based on FAERS database (Jing et al., 2020). Furthermore, the predictive performance of the combination of *LCP1* and *AGDPGK* was validated in an independent patient-level validation cohort, demonstrating clinical utility in predicting irAEs in lung cancer (Jing et al., 2020).

As a pervasive and vital post-transcriptional regulatory mechanism, alternative splicing (AS) is partly responsible for transcript variation and proteome diversity, with more than 95% of transcribed human genes undergoing splicing (Pan et al., 2008). It is known that dysfunction of splicing processes contributes to cancer progression and therapy resistance, but the relevance of alternative splicing for the pathogenesis of irAE is still poorly understood. To fill this gap, we combine the power of real-world pharmacovigilance and omics data to determine if alternative splicing characteristics are associated with ICI-induced irAEs. The biomarker potential of AS for irAEs in cancer immunotherapy is also explored in this study. Our study provides a unique perspective on the links between AS and irAEs.

## Methods

### Data Collection and Pre-Processing

We considered four distinct classes of splicing characteristics: 1) The expression abundances of splicing factors: We downloaded high-quality gene expression profiles of The Cancer Genome Atlas (TCGA) samples from UCSC Xena (Goldman et al., 2021). A total of 404 known and potential auxiliary splicing factors obtained from Seiler et al. (2018) were subjected to downstream analysis. The splicing factors with low abundances were removed (the max TPM value >10, and the median TPM value >2). The median values of each splicing factor were calculated for each cancer type and were in the form of log_2_ (Transcripts Per Million [TPM]+0.001). 2) Descriptors of splicing levels, including splicing load and splicing frequency. The profiles of all five major alternative splicing types identified in TCGA samples were downloaded from the Genomic Data Commons (Kahles et al., 2018). To build a confident set of AS events, we implemented a series of stringent filters. AS events detected in more than ten samples were included in the subsequent analysis. We then filtered out splicing events with PSI <0.05, or PSI >0.95 to reduce the incidence of false positives (Pimentel et al., 2014). Splicing load was determined using filtered AS events of TCGA samples. We counted the number of all AS events per given sample as the total splicing load. The median values of the total splicing load were calculated for each cancer type. Towards obtaining a more detailed view of splicing load, we considered splicing load for each AS type separately. We further classified AS events into four categories, with highly included (PSI >80%), mid-included (40% < PSI <80%), mid-excluded (20% < PSI <40%) and highly excluded events (PSI <20%), as described in a previous study (Agirre et al., 2021). Splicing load for each AS category was calculated as described above, and the median values for each cancer type were obtained. Splicing frequency for the individual gene was defined as the proportion of samples with AS events detected. 3) Neoantigen-related variables, including neojunction load and autoantigen load. For neojunction load, the number of neojunctions per sample for TCGA cancer types was obtained from the Genomic Data Commons (Kahles et al., 2018), and the median values of neojunction load were calculated for each cancer type. We then counted the number of AS events of autoantigen genes per given sample to calculate the splicing load of autoantigen genes. Autoantigen genes were retrieved from the AAgAtlas database (Wang et al., 2016). 4) The expression abundances of splicing isoforms: We collected splicing isoform expression profiles of TCGA samples from UCSC Xena (Goldman et al., 2021). Low abundance isoforms were removed (the max TPM value >10, and the median TPM value >2). The median values of each isoform were calculated for each cancer type and converted info log_2_ (TPM+0.001). Additionally, we considered other factors that have been previously reported to be potentially associated with anti-PD1/PD-L1 response, including 36 variables belonging to three distinct classes (Lee and Ruppin, 2019).

To obtain unbiased estimates of clinical risk of ICI-associated irAEs, reporting odds ratios (RORs), a measure of disproportionality used in pharmacovigilance databases, were obtained from Jing et al. (2020). IrAE RORs were calculated as follows: Individual safety reports submitted between July 2014 and June 2019 were retrieved from the FAERS database. We considered only reports for which anti-PD-1/PD-L1 agents (cemiplimab, nivolumab, pembrolizumab, atezolizumab, avelumab, and durvalumab) were the suspected cause of adverse events. Disproportionality analysis (Bate and Evans, 2009) was then performed to estimate irAE RORs by using the entire database as the comparator. We considered cancer types only for which there were at least 1,000 cases receiving ICIs therapy reported in FAERS. These cancer types include Bladder Urothelial Carcinoma [BLCA], Breast invasive carcinoma [BRCA], Cervical squamous cell carcinoma and endocervical adenocarcinoma [CESC], Cholangiocarcinoma [CHOL], Colon adenocarcinoma [COAD], Esophageal carcinoma [ESCA], Glioblastoma multiforme [GBM], Head and Neck squamous cell carcinoma [HNSC], Liver hepatocellular carcinoma [LIHC], Lung adenocarcinoma [LUAD], Lung squamous cell carcinoma [LUSC], Mesothelioma [MESO], Ovarian serous cystadenocarcinoma [OV], Pancreatic adenocarcinoma [PAAD], Prostate adenocarcinoma [PRAD], Sarcoma [SARC], Skin Cutaneous Melanoma [SKCM], Stomach adenocarcinoma [STAD], Uterine Corpus Endometrial Carcinoma [UCEC].

### Identification of Potential irAE Biomarkers by Combining Alternative Splicing Characteristics and Pharmacovigilance Data

For the scenarios with fewer observations than features, as in the case of our study, the advanced algorithms could have an inflated type I error and subsequently cause more false positives, such as Ridge, LASSO, and Elastic Net regression (Wu and Ma, 2015; Kirpich et al., 2018; Jing et al., 2020). Therefore, we employed an approach to identify splicing characteristics most strongly associated with irAE risk, as described in a previous study (Lee and Ruppin, 2019). We first measured the correlation between single variable and irAE ROR to identify potential biomarkers for irAE risk. Subsequently, we performed a standard regression analysis with leave-one-out cross-validation in predicting irAE ROR from bivariate and trivariate linear-regression models using caret package (Kuhn, 2008). The predictive performance was evaluated based on Spearman rank correlation coefficient (*Rs*) and unexplained variance (1−*Rs* ^2^). For comparison, the goodness of fit between different models was assessed by a log-likelihood ratio test using lmtest package (Hothorn et al., 2015). Statistical significance was defined as *p* < 0.05.

### Functional Annotation and Enrichment Analysis

The parental genes of splicing isoforms were subjected to biological function annotation and enrichment analysis using clusterProfler package (Yu et al., 2012) and KOBAS online tool (Xie et al., 2011) The level of significance was defined as q value or corrected *p* value <0.05. GO term complexity was reduced by measuring semantic similarity using rrvgo (Sayols, 2020). Gene set enrichment analysis (GSEA) (Subramanian et al., 2005) was employed to dissect the immune-related and autoimmune gene sets significantly associated with irAE-related genes. Autoimmune gene sets were derived from Gene and Autoimmiune Disease Association Database (GAAD) (Lu et al., 2018), and immune-related gene sets were collected from the literature (Fischer and Rausell, 2016) and ImmuneDB (Rosenfeld et al., 2017) (Supplementary Table S1). All genes were ranked concerning their correlation with irAE ROR. Gene set variation analysis (GSVA) (Hänzelmann et al., 2013) was used to obtain the GSVA enrichment scores of each sample against immune-related gene sets. Gene sets were considered significantly enriched if their adjusted *p*-value was <0.05.

## Results

### The Association Between irAEs and Alternative Splicing Characteristics

**FIGURE 1:**
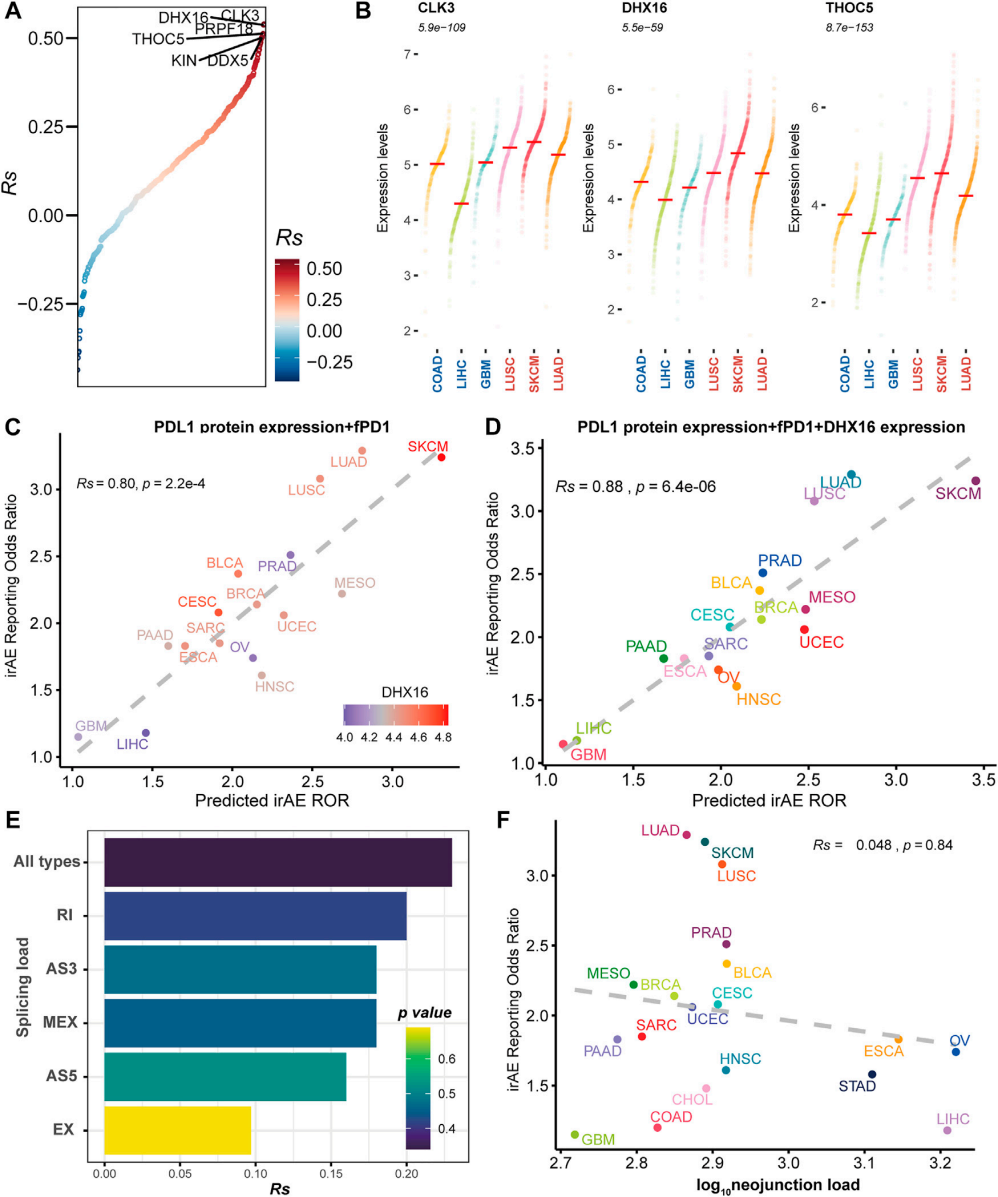
The association between irAE and alternative splicing characteristics. **(A)** Spearman correlation between splicing factor expression and irAE ROR. All splicing factors are ordered by correlations with irAE ROR. The splicing factors with *Rs* > 0.5 are labeled by name. **(B)** Expression distribution of the top three significantly irAE correlated splicing factors in high- and low-irAE ROR cancer types, including *CLK3*, *DHX16*, and *THOC5*. Each dot represents log_2_ (TPM+0.001) transformed expression level of each splicing factor in a single sample. The median of the expression level of each splicing factor for each cancer type is indicated by a horizontal red line. The Wilcox-test *p* value, comparing the difference of expression levels between high- and low- irAE ROR groups, is delineated at the top. **(C)** Combined effect of PD-L1 protein expression + the fraction of high PD-1 messenger RNA expression samples (fPD1) bivariate model (*Rs* = 0.80, *p* = 2.2e-04). The dashed line represents the linear fit. Spearman’s correlation coefficient (*Rs*) was calculated between predicted and observed irAE ROR. *Rs* and the corresponding *p* value are shown at the top-left of the figure. The regression formula for irAE ROR is −1.80 × PD-L1 protein expression +3.97 × fPD1 + 1.73. *DHX16* expression in each cancer type is color-coded. **(D)** Combined effect of PD-L1 protein expression + fPD1 + *DHX16* expression trivariate model (*Rs* = 0.88; *p* = 6.4e-06). The regression formula for irAE ROR is −1.77 × PD-L1 protein expression +2.87 × fPD1 + 0.83 × *DHX16* expression −1.74. **(E)** Spearman correlation between irAE ROR and splicing load of each splicing mode. The bar represents *Rs*, whereas the color indicates *p* value. **(F)** Spearman correlation between neojunction load and irAE ROR. The *x*-axis indicates the neojunction load across 19 cancer types, defined as the median number of the total number of neojunction. AS3 alternative 3′ splice site, AS5 alternative 5′ splice site, EX exon skipping, MEX mutually exclusive exons, RI intron retention. irAE immune-related adverse events, ROR reporting odds ratio, LUAD lung adenocarcinoma, SKCM skin cutaneous melanoma, LUSC lung squamous cell carcinoma, PRAD prostate adenocarcinoma, BLCA bladder urothelial carcinoma, MESO mesothelioma, BRCA breast invasive carcinoma, CESC cervical squamous cell carcinoma and endocervical adenocarcinoma, UCEC uterine corpus endometrial carcinoma, SARC sarcoma, ESCA esophageal carcinoma, PAAD pancreatic adenocarcinoma, OV ovarian serous cystadenocarcinoma, HNSC head and neck squamous cell carcinoma, STAD stomach adenocarcinoma, CHOL cholangiocarcinoma, COAD colon adenocarcinoma, LIHC liver hepatocellular carcinoma, GBM glioblastoma multiforme.

To delineate the relationship between immune-related adverse events and alternative splicing, we studied 19 cancer types for which alternative splicing characteristics from 6906 TCGA patients and irAE reporting odds ratio (ROR) were available. We first focused on splicing factors reported to modulate alternative splicing. We evaluated the association of the expression levels of splicing factors and irAE ROR. We identified twelve potential predictors with *Rs* within the range of [0.46, 0.54] (Supplementary Table S2 and Figure 1A). Among them, slightly higher correlations were observed in CDC like kinase 3 (*CLK3*), ATP-dependent RNA helicase (*DHX16*), and THO Complex 5 (*THOC5*) (Figure 1A). Compared to cancer types with low ROR, these predictors were more abundant in high ROR ones (Figure 1B).

We then asked whether these splicing factors could enhance the prediction performance of other predictive factors, which were significantly correlated to irAE risk (Supplementary Figure S1). The combined PD-L1 protein expression and the fraction of high PD-1 messenger RNA expression samples (fPD1) model was highly correlated with irAE ROR (*Rs* = 0.80, *p* = 2.2e-04), explaining more than 64% of the irAE ROR variance observed across different tumor types. Notably, most of the cancer types that showed higher irAE ROR than predicted by the combined PD-L1 protein expression and fPD1 model had higher *DHX16* expression abundances, whereas those showing lower than bivariate model-based predicted irAE risk had lower *DHX16* expression levels (Figure 1C). Accordingly, trivariate model combining PD-L1 protein expression, fPD1 and *DHX16* expression markedly enhanced the irAE prediction (*Rs* = 0.88, *p* = 6.4e-06; Figure 1D) with a significant log-likelihood model improvement compared with bivariate model (*p* < 2.2e-16). The multicollinearity assessment among the variables showed that no variable exceeded the critical values of VIF >4 (Supplementary Figure S2). These results suggested that these splicing factors can hold promise as biomarkers for irAEs.

Neoantigens stemmed from TMB have been suggested to contribute to irAE development (Bomze et al., 2019). Given that alternative splicing is an important source of potential neoantigens (Lee and Ruppin, 2019), we hereby assessed the predictive potential of splicing load and neojunciton load for irAE risk. No significant correlations existed between irAE risk and splicing load, as well as neojunction load (Figures 1E,F and Supplementary Figure S3). Increased predictive performance was detected in the bivariate model combining neojunction load and mutational burden, albeit the statistical significance cutoff was not met (Supplementary Figure S4). The splicing load of autoantigen genes was also irrelevant to irAE ROR (Supplementary Figure S5). These observations, although unexpected, led us to consider that the role of alternative splicing in irAE pathogenesis may be predominantly mediated through impacting a portion of protein function, rather than releasing excess altered antigens.

### Statistic Association of Gene Splicing Frequency and irAE ROR

**FIGURE 2:**
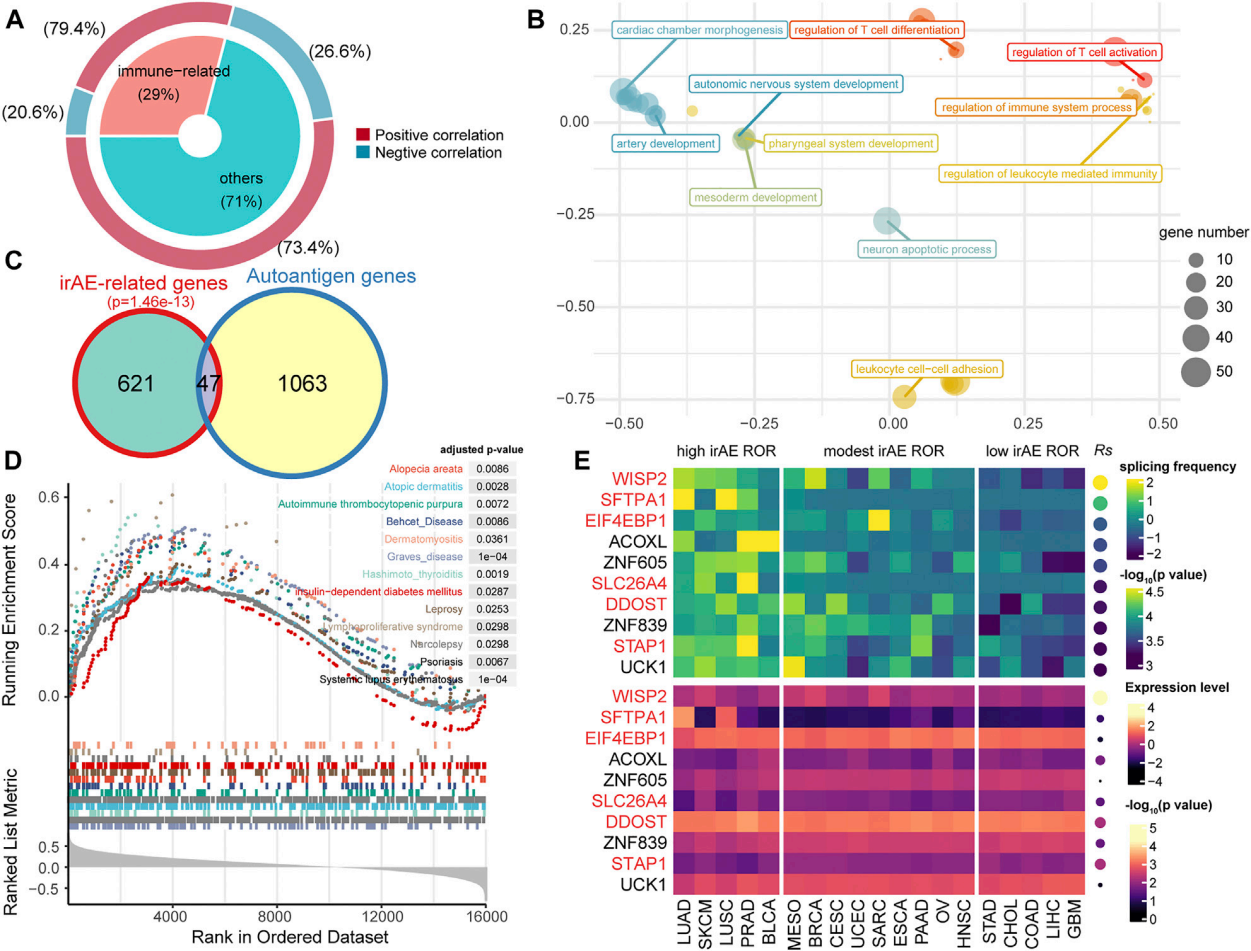
Statistic correlation of splicing frequency and irAE risk across 19 cancer types. **(A)** Overview of irAE-related genes detected by correlation analysis between irAE ROR and splicing frequency of individual gene. The donut plot provides information about the proportion of immune-related genes in all irAE-related genes. **(B)** Functional enrichment analysis for highly positively correlated genes. The scatter plot displays the enriched GO terms. GO term complexity was reduced by measuring semantic similarity using rrvgo. Distances between points represent the similarity between terms. The size of the point represents the number of genes the GO term contains. **(C)** Venn plot of irAE-related genes and autoantigen genes. The *p* value of the hypergeometric test is delineated at the top. **(D)** Gene set enrichment analysis (GSEA) using the autoimmune disease gene sets from GAAD. The input to GSEA pre-ranked module was a ranked list of genes determined by *Rs* across all genes. **(E)** Splicing frequency and expression abundance of the top ten genes significantly correlated with irAE ROR across multiple cancer types. The genes involved in immune response processes were highlighted in red color. Columns represent cancer types. The leftmost panel corresponds to cancer types with high irAE ROR, the middle panel to cancer types with modest irAE risk, and the right panel to cancer types with low irAE ROR. The top panel indicates the splicing frequency of genes. Rows are sorted according to *Rs*. *Rs* was calculated from the correlation analysis between the splicing frequency of genes and irAE ROR. The bottom panel shows the expression abundance of each gene, in which *Rs* was calculated from the expression level of each gene and irAE ROR.

We used splicing frequencies of genes as indicators and evaluated the association between these indicators and irAE risk. Positive or negative hits were defined as irAE-related genes (*p* < 0.05). We identified 668 potential predictors based on correlation analysis (Supplementary Table S3). Notably, the preponderance of irAE-related genes identified was positively correlated with irAE risk, with a smaller number was inversely correlated to irAE ROR (Figure 2A). The genes highly positively associated with irAE (*Rs* > 0.5, *p* < 0.05) were significantly enriched in immune response processes, including leukocyte mediated immunity, regulation of immune system process, as well as T-cell differentiation and activation (Figure 2B). Strikingly, autoantigen genes were preferentially over-represented in irAE-related genes (*p* = 1.46e-13, hypergeometric test; Figure 2C), suggesting a close link between autoimmunity and irAE development. Gene set enrichment analysis reinforced the above findings, in which irAE ROR positively correlated genes were significantly enriched in autoimmune disease gene sets (Figure 2D). These results suggested that alternative splicing might contribute to the connection between autoimmunity and irAEs development.

The splicing frequencies of the top ten irAE ROR correlated genes were depicted by heatmap (Figure 2E). More than half of them were reported to be involved in immune response processes (Figure 2E). Of particular interest, Wnt-1 induced secreted protein-2 (*WISP2/CCN5*), which is involved in inflammation response and autoimmune disease (Tanaka et al., 2005; Macdonald et al., 2021), achieved the highest correlation coefficient (*Rs* = 0.81, *p* = 2.82e-5; Figure 2E). In addition, the expression levels of most predictors were independent of irAE ROR (Figure 2E), indicating extraordinary predictive values of gene splicing frequency for irAE risk.

### Comprehensive Identification for Potential irAE Biomarkers on Isoform-Level

**FIGURE 3:**
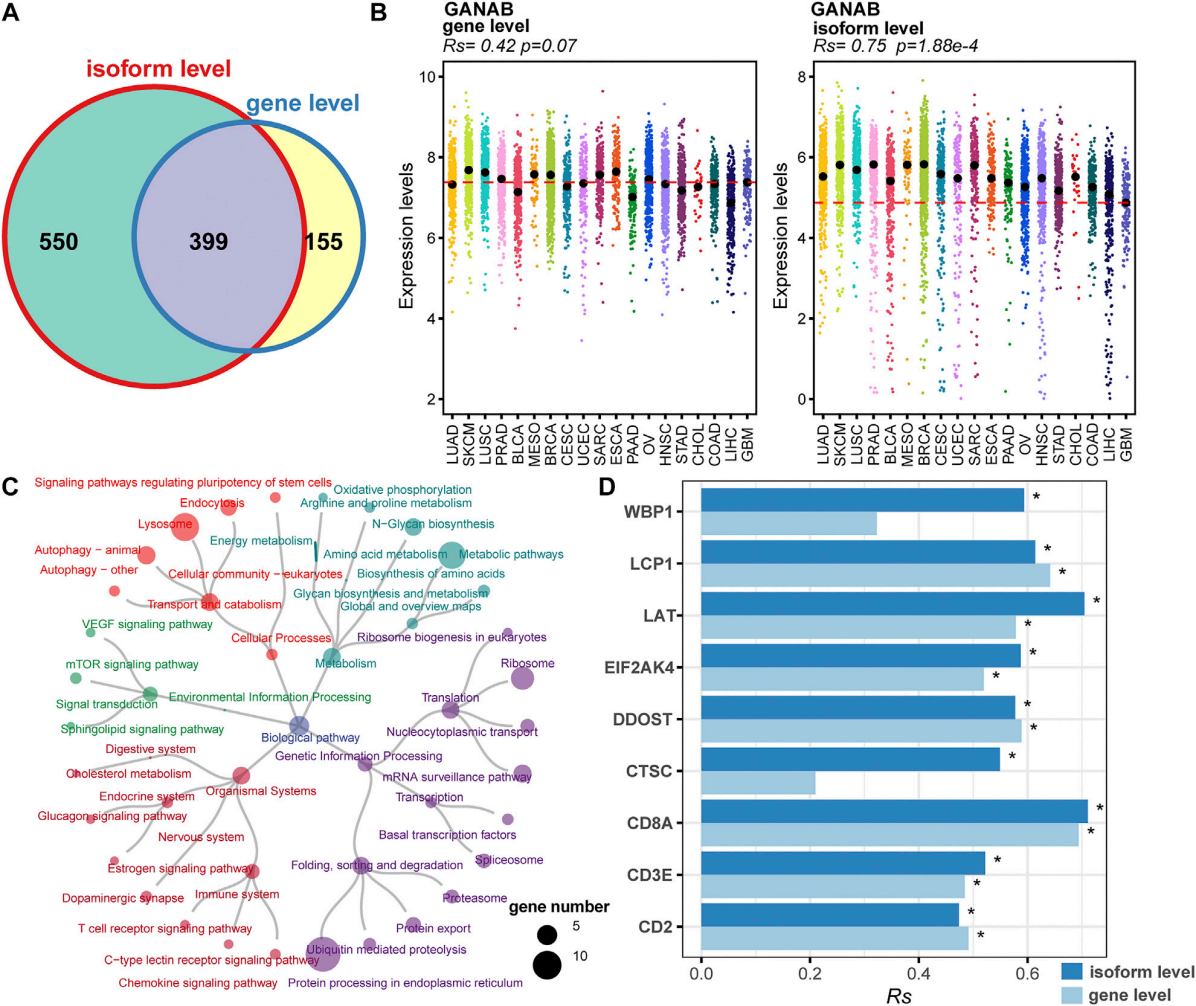
Identification of irAEs-related splicing isoforms. **(A)** Venn plot of irAEs-related genes identified on gene- and isoform-level, respectively. **(B)** Spearman correlation between irAE ROR and *GANAB* expression on gene and isoform level. *Rs* and the corresponding *p* value are shown at the top-left of the figure. Strip plots show expression distribution of *GANAB* on gene **(left)** and isoform level **(right)**. Each column represents a cancer type. Each dot corresponds to log_2_ (TPM+0.001) transformed expression value of the selected gene in one sample on gene- and isoform-level, respectively. The dashed lines display the median of GANAB expression in GBM on gene- and isoform-level, respectively. **(C)** Pathway enrichment analysis for positive correlated splicing isoforms to irAE. The pathways are colored according to pathway hierarchy. The number of parental genes is shown by dot size. **(D)** Spearman correlation between irAE ROR and splicing isoforms involved in T cell activation and T cell-mediated cytotoxicity. * indicates significant correlation (*p* value <0.05). The bar represents *Rs*, whereas the color indicates *Rs* is calculated at gene or isoform level.

Splicing isoforms produced by alternative splicing encompass the information about expression abundance and differences in exon inclusion or exclusion, which may be valuable resources to derive surrogate biomarkers for irAEs. Thus, we detected and characterized the relationship between the expression of splicing isoforms and irAE ROR using pan-cancer data. In total, 1,131 splicing isoforms derived from 949 genes, were significantly associated with irAE ROR (Figure 3A). Particularly, 550 genes corresponding to 618 splicing isoforms were significantly correlated with irAE risk only on isoform-level (Figure 3A), such as *GANAB, ELP2,* and *MTCH1*, which would be overlooked by standard gene-level analysis. As exhibited in Figure 3B, *GANAB-201* was the strongest positive correlate of irAE ROR (*Rs* = 0.75; *p* = 1.88e-4), but the expression level of its parental gene was unrelated to irAE ROR (Figure 3B).

We applied pathway enrichment analysis to analyze irAE-related splicing isoforms. Enriched pathways were divided into five groups based on functional hierarchy. Significantly, immune-related pathways were highly enriched, such as T cell receptor signaling pathway, chemokine signaling pathway, and C-type lectin receptor signaling pathway (Figure 3C). Specifically, several genes involved in T cell activation and T cell mediated cytotoxicity were significantly correlated with irAE ROR on isoform-level (Figure 3D), such as *WBP1*, *LAT*, *EIF2AK4*, and *CTSC*, suggesting alternative splicing may mediate irAE development by fine-tuning gene expression. Other pathways related to metabolism, genetic information processing, and signal transduction were also strongly enriched (Figure 3C), indicating that there is likely substantial complexity underlying irAE development, although currently analyses remain do not offer proof of causal relationships.

**FIGURE 4:**
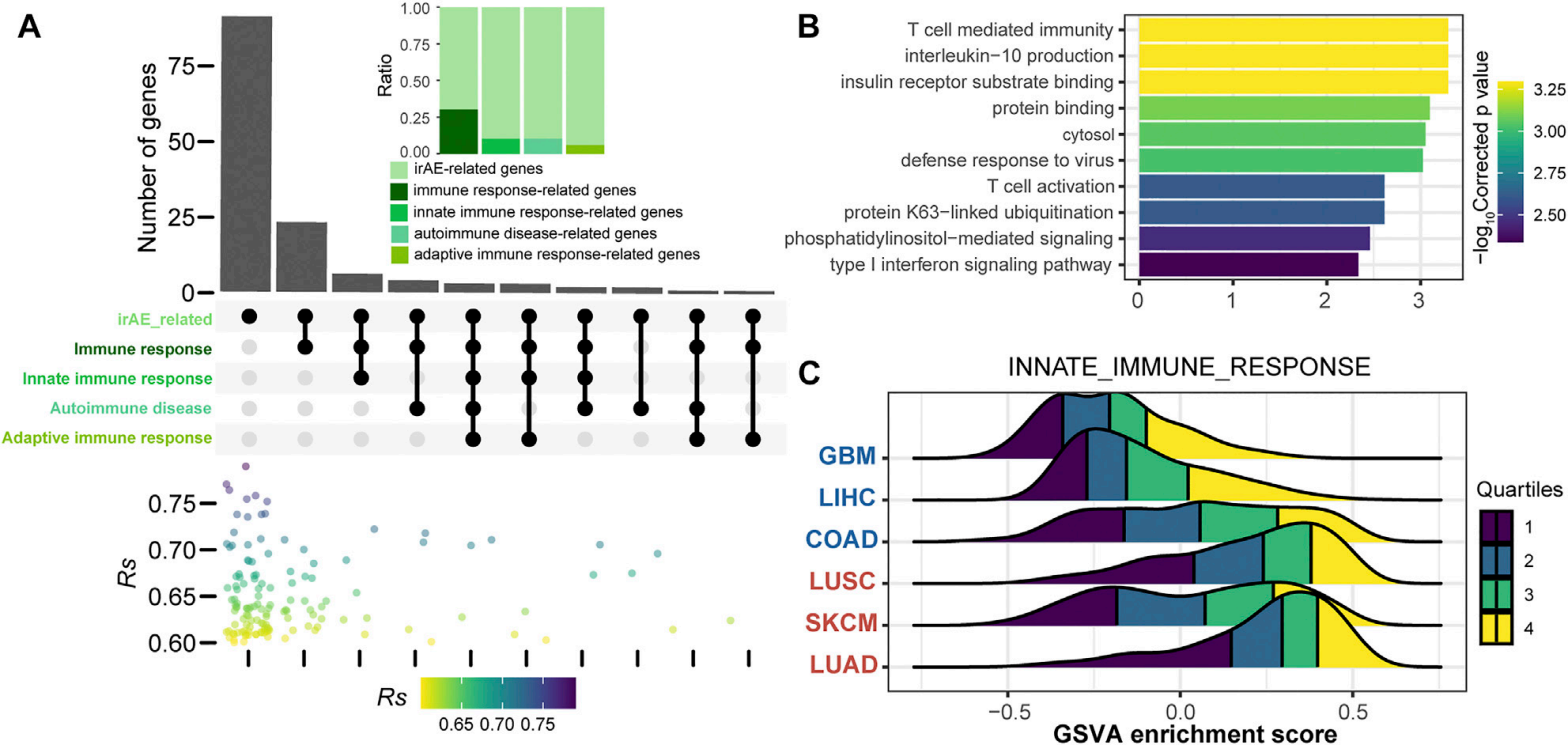
Functional characteristics of irAE ROR significantly correlated splicing isoforms. **(A)** The intersections of parental genes of highly positively irAEs-related splicing isoforms (*Rs* > 0.6, *p* value < 0.05) with immune-related and autoimmune disease gene sets. The color dot plot shows *Rs* values of irAEs-related splicing isoforms in each corresponding subset. The upper inset shows the proportion of irAE-related splicing isoforms annotated into the corresponding gene sets. **(B)** Functional enrichment analysis of irAEs-related splicing isoforms associated with innate immune response (top 10). Bar plot shows -log_10_Corrected *p* values of significantly enriched pathways. **(C)** GSVA enrichment scores of innate immune response in patients with high and low irAE risk.

To further explore the function of irAE-related splicing isoforms, three curated gene sets related to innate and adaptive immune responses, as well as autoimmune diseases were used to characterize the immune phenotype (Figure 4A). A relatively large proportion of highly positively correlated splicing isoforms were involved in innate immune response and autoimmune diseases (Figure 4A). Function enrichment analysis revealed innate immune response-related predictors participated in important immune response processes, such as T cell mediated immunity, interleukin-10 production, and T cell activation (Figure 4B). GSVA analysis further revealed that innate immune response as major biology process activated in cancer types with high irAE risk (Figure 4C). Moreover, the adaptive immune response also exhibited a significant activity difference between high and low irAE ROR cancer types (Supplementary Figure S6). These results suggested alternative splicing might contribute to irAE pathogenesis via coordinating the activation of both innate and adaptive immunity.

Previous studies have suggested that irAEs are promising predictors for the efficacy of ICIs (Zhong et al., 2021). Therefore, we next asked whether these splicing isoforms could not only reflect irAE risk but also have the potential of predicting response to ICIs. The result showed that only 30 splicing isoforms were also indicators of ICIs response based on the associations with the objective response rate (ORR) of 16 cancer types obtained from Lee and Ruppin (2019) (Supplementary Table S4).

### Construction of Regression Model to Predict irAE Risk

**FIGURE 5:**
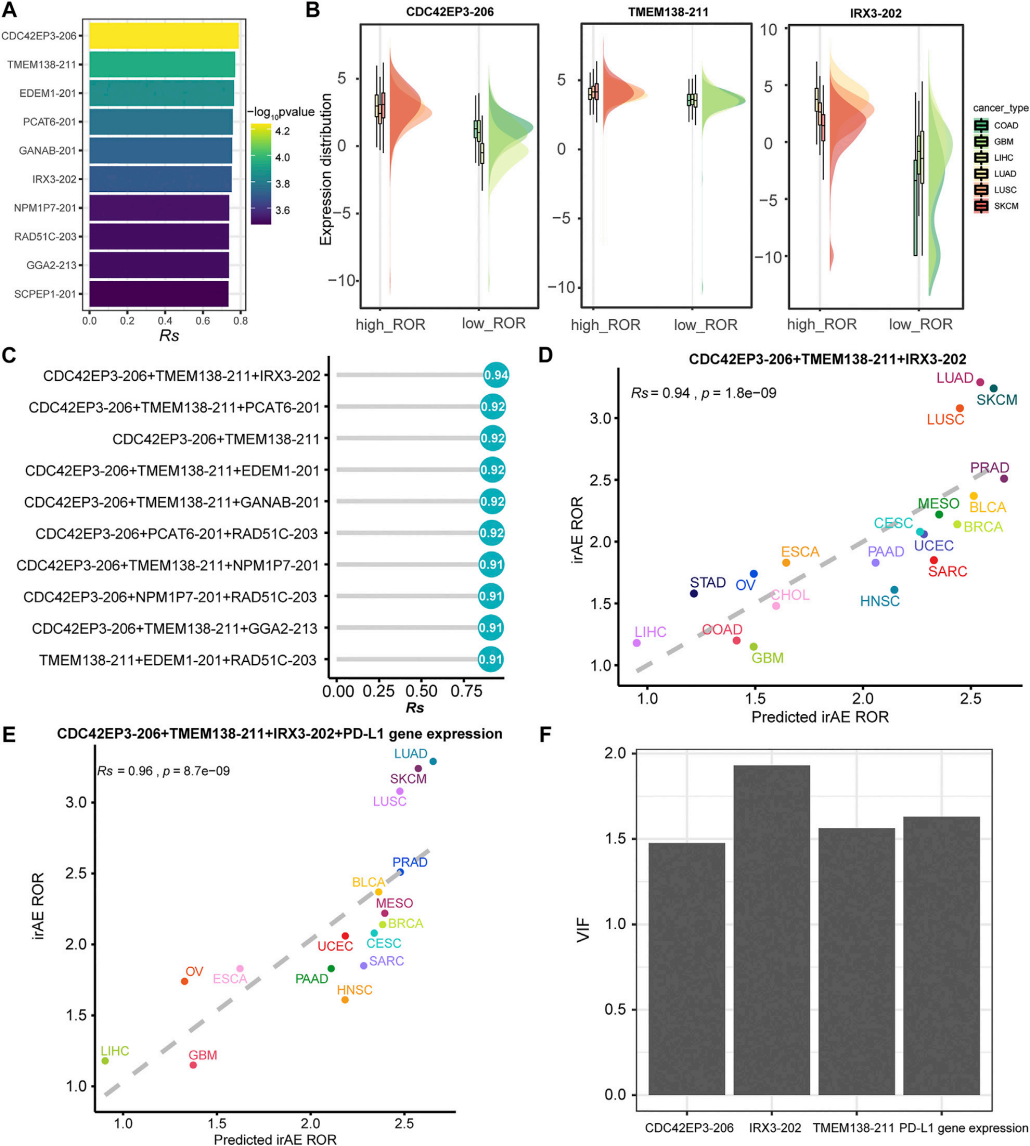
Regression analysis for splicing isoforms to predict irAE risk. **(A)** The top ten splicing isoforms significantly correlated with irAE ROR across multiple cancer types. The bar represents *Rs*, and the color indicates the corresponding *p* value. **(B)** Expression distribution of representative splicing isoforms positively correlated to irAE in different TCGA cohorts. Boxplots represent log_2_ (TPM +0.001) values for splicing isoforms. Within each TCGA cohort, the bottom and top of the boxes are the 25th and 75th percentiles (interquartile range), and the thick line represents the median value. The whiskers encompass 1.5 times the interquartile range. **(C)** Comparison of performance of bivariate and trivariate models in predicting irAE for all combinations of the top ten irAE ROR significantly correlated splicing isoforms. *Rs* was calculated between predicted and observed irAE ROR. **(D)** Combination of CDC42EP3-206, TMEM138-211, and IRX3-202 to predict irAE risk. The dot color represents the cancer type. The dashed line represents the linear fit. **(E)** Combination of CDC42EP3-206, TMEM138-211, IRX3-202, and PD-L1 gene expression to predict irAE risk. **(F)** Multicollinearity assessment of the models fitted using the Variance Inflation Factor (VIF).

We further sought to identify more powerful predictive models that can be more easily translated into clinical practice. The top ten irAE ROR significantly correlated splicing isoforms were utilized for building the irAE ROR predictions (Figure 5A). These predictors were predominantly upregulated in cancer types with high irAE ROR compared to low irAE ROR cancer types (Figure 5B). CDC42EP3-206 was the most predictive of irAE risk across cancer types (*Rs* = 0.79, *p* = 5.8e-05), followed by TMEM138-211 (*Rs* = 0.77, *p* = 1.1e-04). Combinations between any two or three of these predictors were then evaluated by Spearman correlation and goodness of fit using the log-likelihood ratio test. Notably, the combination of CDC42EP3-206 and TMEM138-211 with most of the other predictors achieved better predictive performance (Figure 5C). Specifically, the CDC42EP3-206 + TMEM138-211 + IRX3-202-based model achieved maximum predictive efficacy (*Rs* = 0.94, FDR = 1.8e-09), explaining more than 88% of the ROR variance observed across different tumor types (Figures 5C,D). We further evaluated the performance of the combinations of CDC42EP3-206 + TMEM138-211 + IRX3-202 with other factors. Finally, we established a combination model composed of CDC42EP3-206, TMEM138-211, IRX3-202, and *PD-L1* gene expression (Figure 5E), with a slightly enhanced prediction performance (*Rs* = 0.96, *p* = 8.7e-09). The relationship assessment between these predictors indicated that no multicollinearity was detected, suggesting the independent prediction for irAE (Figure 5F).

## Discussion

Cancer treatment by immune checkpoint inhibitors holds promise for cancer therapy. With the growing use of ICIs, unpredictable irAEs exhibit a significant increase, and become a major obstacle for the optimal application of ICIs in cancer therapy. The predictive value of alternative splicing for prognosis and drug resistance has been suggested by several studies. However, the predictive potential of alternative splicing for irAE has not been clarified. To deeply understand the relevance of splicing modulation for irAE development, we systematically investigated the relationships between alternative splicing characteristics and irAE risk by integrating pharmacovigilance and molecular data.

Emerging evidence expounds the roles of splicing factors in cancer and immunity (Yang et al., 2021). The function of splicing factors in irAE development has not been described. In the present study, twelve splicing factors were identified to be significantly correlated with irAE risk (Supplementary Table S2), suggesting predictive value for irAE. Indeed, adding *DHX16* to the bivariate PD-L1 protein expression-fPD1 model led to a trivariate regression model with a significantly improved accuracy and decreased the unexplained variance from 0.36 (1–0.80^2^) to 0.23 (1–0.88^2^) (Figure 2D). *DHX16* is linked to several malignant and autoimmune diseases, and its role in innate immunity has been documented (Gencheva et al., 2010; Hage et al., 2019). Our observation provides new evidence for splicing factors as another instrument for the regulation of irAEs.

By considering alternative splicing-derived, in addition to mutant-derived, peptides as potential antigens, we interrogated the associations between irAE risk and the descriptors associated with alternative splicing-derived neoantigens, including the total splicing load, neojunction load, and splicing load of autoantigen genes. We failed to find a correspondence between irAE risk and these descriptors (Figures 1E,F and Supplementary Figures S3–S5). The above evidence indicated that alternative splicing seems to affect irAE development by driving functional effects on particular genes rather than increasing overall splicing diversity to release excessive altered antigens. It is quite possible that a large fraction of the increased splicing diversity is a passenger due to disrupted splicing machinery in cancer cells, which often leads to a lower accuracy of splicing (Pickrell et al., 2010).

Alternative splicing is becoming increasingly recognized as an important mechanism for the generation of structural and functional diversity in proteins, which can alter protein function, and even remodel protein-protein interaction networks. Our analysis revealed a significant correlation between the ROR of reporting an irAE during ICIs therapy and the corresponding gene splicing frequency across multiple cancer types. The prevalence of alternative splicing was found to be highest in genes related to immune response processes, especially in T cell differentiation and activation (Figure 2B). Moreover, nine splicing isoforms involved in T cell activation and T cell mediated cytotoxicity were significantly associated with irAE risk (Figure 3C). Our findings align well with the evidence of T cell response in irAE lesions. Our results also revealed significant enrichment of irAE-related splicing isoforms in signaling pathways that have been previously associated with irAEs, including mTOR and JAK/STAT signaling pathways (Figure 3C, Supplementary Table S4). The mTOR signaling pathway has an important role in the modulation of both innate and adaptive immune responses (Powell et al., 2012). Aberrant activation of mTOR signaling has been known to contribute to the pathogenesis of autoimmune disorders and cancer (Perl, 2015). Presumably, mTOR signaling has a similar pathogenic role and potential therapeutic target in irAEs. The mTOR inhibitor in combination with anti-PD-1 therapy did indeed maintain allograft tolerance without compromising anti-tumor efficacy (Esfahani et al., 2019). Similar to mTOR signaling, the JAK/STAT pathway has been implicated in the pathogenesis of autoimmune states and irAEs (Esfahani et al., 2020), and the inhibition of JAK/STAT signaling has led to remarkable remissions in the setting of autoimmune disorders (Lee et al., 2014; Sandborn et al., 2017). The mTOR and JAK/STAT signaling pathways have emerged as promising therapeutic targets for the treatment of irAEs. Besides, irAE-related predictors were also strongly enriched in other pathways related to metabolism, genetic information processing, and cellular processes (Figure 3C), indicating the complexity of the biological basis underlying irAE development. However, the impacts of these pathways for irAE pathogenesis have yet to be fully elucidated. Further research into the roles of these predictors during checkpoint blockade may be critical for developing combination therapies to uncouple the efficacy and toxicity of ICIs and overcome irAE risk.

Furthermore, a part of predictors for irAEs identified in the present study was related to autoimmune diseases (Figure 2D and Figure 4A). Specifically, numerous autoantigen genes with high splicing frequency were significantly correlated with irAE (Figure 2C). Therefore, we speculate that splicing of autoantigen genes may, to some extent, be related to irAE development. Increased noncanonical splicing of autoantigen genes augments the probability of confronting the immune system with untolerized epitopes and eventually leads to irAEs (Ng et al., 2004).

The current evidence points towards a crucial role of the innate immune system in potentially driving irAEs (Esfahani et al., 2020), as demonstrated in this study (Figure 4). Innate immune cells can mediate irAE development likely both in cooperation with and independent of adaptive immune cells (Lee et al., 2021). Studies have reported associations of irAEs with the recruitment of CD14^+^CD16^+^ monocytes (Curry et al., 2019), the presence of eosinophilia (Kizawa et al., 2019), increased neutrophil/lymphocyte ratio (NLR) (Drobni et al., 2020), as well as NK cell-mediated antibody-dependent cell-mediated cytotoxicity (ADCC) reactions (Kelly et al., 2018). In addition to the innate immunity, seven genes related to the adaptive immune response also underwent alternative splicing and that were highly correlated with irAE risk (Figure 4A). Deciphering the connections between innate and adaptive immunity and adverse reactions from the perspective of alternative splicing holds promise for a better understanding of both processes.

To our knowledge, this is the first systematic evaluation of the relationship between alternative splicing characteristics and the risk of developing irAEs. Despite being preliminary, we envision that alternative splicing characteristics may represent meaningful biomarkers for irAEs and immunotherapy response in clinical practice. Understanding the factors that contribute to irAE development may help prevent and treat irAEs in patients undergoing ICIs. To establish alternative splicing characteristics as new biomarkers for irAE development, further studies will be needed to validate our findings in a larger, independent cohort. It would be of significant interest to test the clinical utility of these predictors in ICI decision-making in a prospective clinical trial.

## Data Availability

Individual safety records can be accessed through FAERS Public Dashboard (https://www.fda.gov/drugs/questions-and-answers-fdas-adverse-event-reporting-system-faers/fda-adverse-event-reporting-system-faers-public-dashboard). The PSI values of AS events detected in TCGA cohorts were downloaded from the Genomic Data Commons (https://gdc.cancer.gov/about-data/publications/PanCanAtlas-Splicing-2018) (Kahles et al., 2018). TPM-normalized gene and isoform expression data were obtained from UCSC Xena browser (https://xenabrowser.net/) (Jing et al., 2020). All the remaining data will be available from the author upon reasonable request.

## References

[B1] Agirre E. Oldfield A. Bellora N. Segelle A. Luco R. (2021). Splicing-associated Chromatin Signatures: a Combinatorial and Position-dependent Role for Histone marks in Splicing Definition. Nat. Commun. 12, 1–16. 10.1038/s41467-021-20979-x 33514745 PMC7846797

[B2] Bagchi S. Yuan R. Engleman E. G. (2021). Immune Checkpoint Inhibitors for the Treatment of Cancer: Clinical Impact and Mechanisms of Response and Resistance. Annu. Rev. Pathol. Mech. Dis. 16, 223–249. 10.1146/annurev-pathol-042020-042741 33197221

[B3] Bate A. Evans S. J. (2009). Quantitative Signal Detection Using Spontaneous ADR Reporting. Pharmacoepidemiol. Drug Saf. 18, 427–436. 10.1002/pds.1742 19358225

[B4] Bomze D. Hasan Ali O. Bate A. Flatz L. (2019). Association between Immune-Related Adverse Events during Anti-PD-1 Therapy and Tumor Mutational Burden. JAMA Oncol. 5, 1633–1635. 10.1001/jamaoncol.2019.3221 31436791 PMC6707013

[B5] Curry J. L. Reuben A. Szczepaniak-Sloane R. Ning J. Milton D. R. Lee C. H. (2019). Gene Expression Profiling of Lichenoid Dermatitis Immune-Related Adverse Event from Immune Checkpoint Inhibitors Reveals Increased CD14+ and CD16+ Monocytes Driving an Innate Immune Response. J. Cutan. Pathol. 46, 627–636. 10.1111/cup.13454 30883858

[B6] Drobni Z. D. Zafar A. Zubiri L. Zlotoff D. A. Alvi R. M. Lee C. (2020). Decreased Absolute Lymphocyte Count and Increased Neutrophil/Lymphocyte Ratio with Immune Checkpoint Inhibitor-Associated Myocarditis. J. Am. Heart Assoc. 9, e018306. 10.1161/JAHA.120.018306 33190570 PMC7763791

[B7] Esfahani K. Al-Aubodah T. A. Thebault P. Lapointe R. Hudson M. Johnson N. A. (2019). Targeting the mTOR Pathway Uncouples the Efficacy and Toxicity of PD-1 Blockade in Renal Transplantation. Nat. Commun. 10, 4712–4719. 10.1038/s41467-019-12628-1 31624262 PMC6797722

[B8] Esfahani K. Elkrief A. Calabrese C. Lapointe R. Hudson M. Routy B. (2020). Moving towards Personalized Treatments of Immune-Related Adverse Events. Nat. Rev. Clin. Oncol. 17, 504–515. 10.1038/s41571-020-0352-8 32246128

[B9] Fischer A. Rausell A. (2016). Primary Immunodeficiencies Suggest Redundancy within the Human Immune System. Sci. Immunol. 1, eaah5861. 10.1126/sciimmunol.aah5861 28783693

[B10] Gencheva M. Kato M. Newo A. N. Lin R. J. (2010). Contribution of DEAH-Box Protein DHX16 in Human Pre-mRNA Splicing. Biochem. J. 429, 25–32. 10.1042/BJ20100266 20423332 PMC3137565

[B11] Goldman M. J. Craft B. Zhu J.-C. Haussler D. (2021). UCSC Xena for the Visualization and Analysis of Cancer Genomics Data. Philadelphia, Pennsylvania, United States: AACR.

[B12] Hage A. Bharaj P. Rajsbaum R. (2019). Induction of Innate Antiviral Immunity through a Novel Pattern Recognition Receptor, the RNA Helicase DHX16, Is Regulated by Unanchored K48-Linked Poly-Ubiquitin Chains. Rockville, Maryland: Am Assoc Immnol.

[B13] Hänzelmann S. Castelo R. Guinney J. (2013). GSVA: Gene Set Variation Analysis for Microarray and RNA-Seq Data. BMC bioinformatics 14, 7–15. 10.1186/1471-2105-14-7 23323831 PMC3618321

[B14] Hothorn T. Zeileis A. Farebrother R. W. Cummins C. Millo G. Mitchell D. (2015). Package ‘lmtest’. Testing Linear Regression Models. Available at: https://cran.r-project.org/web/packages/lmtest/lmtest.pdf .

[B15] Jing Y. Liu J. Ye Y. Pan L. Deng H. Wang Y. (2020). Multi-omics Prediction of Immune-Related Adverse Events during Checkpoint Immunotherapy. Nat. Commun. 11, 4946–4947. 10.1038/s41467-020-18742-9 33009409 PMC7532211

[B16] Johnson D. B. Balko J. M. Compton M. L. Chalkias S. Gorham J. Xu Y. (2016). Fulminant Myocarditis with Combination Immune Checkpoint Blockade. N. Engl. J. Med. 375, 1749–1755. 10.1056/NEJMoa1609214 27806233 PMC5247797

[B17] Kahles A. Lehmann K. V. Toussaint N. C. Hüser M. Stark S. G. Sachsenberg T. (2018). Comprehensive Analysis of Alternative Splicing across Tumors from 8,705 Patients. Cancer cell 34, 211–224. 10.1016/j.ccell.2018.07.001 30078747 PMC9844097

[B18] Kelly K. Infante J. R. Taylor M. H. Patel M. R. Wong D. J. Iannotti N. (2018). Safety Profile of Avelumab in Patients with Advanced Solid Tumors: a Pooled Analysis of Data from the Phase 1 JAVELIN Solid Tumor and Phase 2 JAVELIN Merkel 200 Clinical Trials. Cancer 124, 2010–2017. 10.1002/cncr.31293 29469949 PMC5947549

[B19] Kerepesi C. Bakacs T. Moss R. W. Slavin S. Anderson C. C. (2020). Significant Association between Tumor Mutational burden and Immune-Related Adverse Events during Immune Checkpoint Inhibition Therapies. Cancer Immunol. Immunother. 69, 683–687. 10.1007/s00262-020-02543-6 32152702 PMC7183506

[B20] Kirpich A. Ainsworth E. A. Wedow J. M. Newman J. R. B. Michailidis G. Mcintyre L. M. (2018). Variable Selection in Omics Data: A Practical Evaluation of Small Sample Sizes. PloS one 13, e0197910. 10.1371/journal.pone.0197910 29927942 PMC6013185

[B21] Kizawa R. Miura Y. Oda Y. Nagaoka Y. Ozaki Y. Kondoh C. (2019). Eosinophilia during Treatment of Immune Checkpoint Inhibitors (ICIs) to Predict Succeeding Onset of Immune-Related Adverse Events (irAEs). Alexandria, Virginia, United States: American Society of Clinical Oncology.

[B22] Kuhn M. (2008). Building Predictive Models in R Using the Caret Package. J. Stat. Softw. 28, 1–26. 10.18637/jss.v028.i05 27774042

[B23] Lee D. J. Lee H. J. Farmer J. R. Reynolds K. L. (2021). Mechanisms Driving Immune-Related Adverse Events in Cancer Patients Treated with Immune Checkpoint Inhibitors. Curr. Cardiol. Rep. 23, 98–12. 10.1007/s11886-021-01530-2 34196833

[B24] Lee E. B. Fleischmann R. Hall S. Wilkinson B. Bradley J. D. Gruben D. (2014). Tofacitinib versus Methotrexate in Rheumatoid Arthritis. N. Engl. J. Med. 370, 2377–2386. 10.1056/NEJMoa1310476 24941177

[B25] Lee J. S. Ruppin E. (2019). Multiomics Prediction of Response Rates to Therapies to Inhibit Programmed Cell Death 1 and Programmed Cell Death 1 Ligand 1. JAMA Oncol. 5, 1614–1618. 10.1001/jamaoncol.2019.2311 31436822 PMC6707018

[B26] Lu G. Hao X. Chen W. H. Mu S. (2018). GAAD: a Gene and Autoimmiune Disease Association Database. Genomics Proteomics Bioinformatics 16, 252–261. 10.1016/j.gpb.2018.05.001 30268934 PMC6205079

[B27] Macdonald I. J. Huang C. C. Liu S. C. Lin Y. Y. Tang C. H. (2021). Targeting CCN Proteins in Rheumatoid Arthritis and Osteoarthritis. Int. J. Mol. Sci. 22, 4340. 10.3390/ijms22094340 33919365 PMC8122640

[B28] Ng B. Yang F. Huston D. P. Yan Y. Yang Y. Xiong Z. (2004). Increased Noncanonical Splicing of Autoantigen Transcripts Provides the Structural Basis for Expression of Untolerized Epitopes. J. Allergy Clin. Immunol. 114, 1463–1470. 10.1016/j.jaci.2004.09.006 15577853 PMC3902068

[B29] Pan Q. Shai O. Lee L. J. Frey B. J. Blencowe B. J. (2008). Deep Surveying of Alternative Splicing Complexity in the Human Transcriptome by High-Throughput Sequencing. Nat. Genet. 40, 1413–1415. 10.1038/ng.259 18978789

[B30] Perl A. (2015). mTOR Activation Is a Biomarker and a central Pathway to Autoimmune Disorders, Cancer, Obesity, and Aging. Ann. N. Y Acad. Sci. 1346, 33–44. 10.1111/nyas.12756 25907074 PMC4480196

[B31] Pickrell J. K. Pai A. A. Gilad Y. Pritchard J. K. (2010). Noisy Splicing Drives mRNA Isoform Diversity in Human Cells. Plos Genet. 6, e1001236. 10.1371/journal.pgen.1001236 21151575 PMC3000347

[B32] Pimentel H. Parra M. Gee S. Ghanem D. An X. Li J. (2014). A Dynamic Alternative Splicing Program Regulates Gene Expression during Terminal Erythropoiesis. Nucleic Acids Res. 42, 4031–4042. 10.1093/nar/gkt1388 24442673 PMC3973340

[B33] Postow M. A. Sidlow R. Hellmann M. D. (2018). Immune-related Adverse Events Associated with Immune Checkpoint Blockade. N. Engl. J. Med. 378, 158–168. 10.1056/NEJMra1703481 29320654

[B34] Powell J. D. Pollizzi K. N. Heikamp E. B. Horton M. R. (2012). Regulation of Immune Responses by mTOR. Annu. Rev. Immunol. 30, 39–68. 10.1146/annurev-immunol-020711-075024 22136167 PMC3616892

[B35] Rosenfeld A. M. Meng W. Luning Prak E. T. Hershberg U. (2017). ImmuneDB: a System for the Analysis and Exploration of High-Throughput Adaptive Immune Receptor Sequencing Data. Bioinformatics 33, 292–293. 10.1093/bioinformatics/btw593 27616708 PMC5254080

[B36] Sandborn W. J. Su C. Sands B. E. D'Haens G. R. Vermeire S. Schreiber S. (2017). Tofacitinib as Induction and Maintenance Therapy for Ulcerative Colitis. N. Engl. J. Med. 376, 1723–1736. 10.1056/NEJMoa1606910 28467869

[B37] Sayols S. (2020). Rrvgo: a Bioconductor Package to Reduce and Visualize Gene Ontology Terms. Available at: https://ssayols.github.io/rrvgo . 10.17912/micropub.biology.000811 PMC10155054 37151216

[B38] Seiler M. Peng S. Agrawal A. A. Palacino J. Teng T. Zhu P. (2018). Somatic Mutational Landscape of Splicing Factor Genes and Their Functional Consequences across 33 Cancer Types. Cell Rep 23, 282–296. 10.1016/j.celrep.2018.01.088 29617667 PMC5933844

[B39] Subramanian A. Tamayo P. Mootha V. K. Mukherjee S. Ebert B. L. Gillette M. A. (2005). Gene Set Enrichment Analysis: a Knowledge-Based Approach for Interpreting Genome-wide Expression Profiles. Proc. Natl. Acad. Sci. U S A. 102, 15545–15550. 10.1073/pnas.0506580102 16199517 PMC1239896

[B40] Subudhi S. K. Aparicio A. Gao J. Zurita A. J. Araujo J. C. Logothetis C. J. (2016). Clonal Expansion of CD8 T Cells in the Systemic Circulation Precedes Development of Ipilimumab-Induced Toxicities. Proc. Natl. Acad. Sci. U S A. 113, 11919–11924. 10.1073/pnas.1611421113 27698113 PMC5081579

[B41] Tanaka I. Morikawa M. Okuse T. Shirakawa M. Imai K. (2005). Expression and Regulation of WISP2 in Rheumatoid Arthritic Synovium. Biochem. Biophys. Res. Commun. 334, 973–978. 10.1016/j.bbrc.2005.06.196 16038875

[B42] Van Hasselt J. G. C. Rahman R. Hansen J. Stern A. Shim J. V. Xiong Y. (2020). Transcriptomic Profiling of Human Cardiac Cells Predicts Protein Kinase Inhibitor-Associated Cardiotoxicity. Nat. Commun. 11, 4809–4812. 10.1038/s41467-020-18396-7 32968055 PMC7511315

[B43] Wang D. Y. Salem J. E. Cohen J. V. Chandra S. Menzer C. Ye F. (2018). Fatal Toxic Effects Associated with Immune Checkpoint Inhibitors: a Systematic Review and Meta-Analysis. JAMA Oncol. 4, 1721–1728. 10.1001/jamaoncol.2018.3923 30242316 PMC6440712

[B44] Wang D. Yang L. Zhang P. Labaer J. Hermjakob H. Li D. (2016). AAgAtlas 1.0: A Human Autoantigen Database. Nucleic Acids Res. 45, D769–D776. 10.1093/nar/gkw946 27924021 PMC5210642

[B45] Wu C. Ma S. (2015). A Selective Review of Robust Variable Selection with Applications in Bioinformatics. Brief Bioinform 16, 873–883. 10.1093/bib/bbu046 25479793 PMC4570200

[B46] Xie C. Mao X. Huang J. Ding Y. Wu J. Dong S. (2011). KOBAS 2.0: a Web Server for Annotation and Identification of Enriched Pathways and Diseases. Nucleic Acids Res. 39, W316–W322. 10.1093/nar/gkr483 21715386 PMC3125809

[B47] Yang H. Beutler B. Zhang D. (2021). Emerging Roles of Spliceosome in Cancer and Immunity. Protein & Cell, 1–21. 10.1007/s13238-021-00856-5 34196950 PMC9232692

[B48] Yu G. Wang L. G. Han Y. He Q. Y. (2012). clusterProfiler: an R Package for Comparing Biological Themes Among Gene Clusters. OMICS 16, 284–287. 10.1089/omi.2011.0118 22455463 PMC3339379

[B49] Zhong L. Wu Q. Chen F. Liu J. Xie X. (2021). Immune-related Adverse Events: Promising Predictors for Efficacy of Immune Checkpoint Inhibitors. Cancer Immunol. Immunother. 70, 2559–2576. 10.1007/s00262-020-02803-5 33576872 PMC10991616

