# Supplementary figures and images for: Pan-Cancer Analysis Reveals Alternative Splicing Characteristics Associated With Immune-Related Adverse Events Elicited by Checkpoint Immunotherapy
Source: Front Pharmacol. 2021 Nov 24;12:797852. doi: 10.3389/fphar.2021.797852 (PMC8652050; doi:10.3389/fphar.2021.797852)

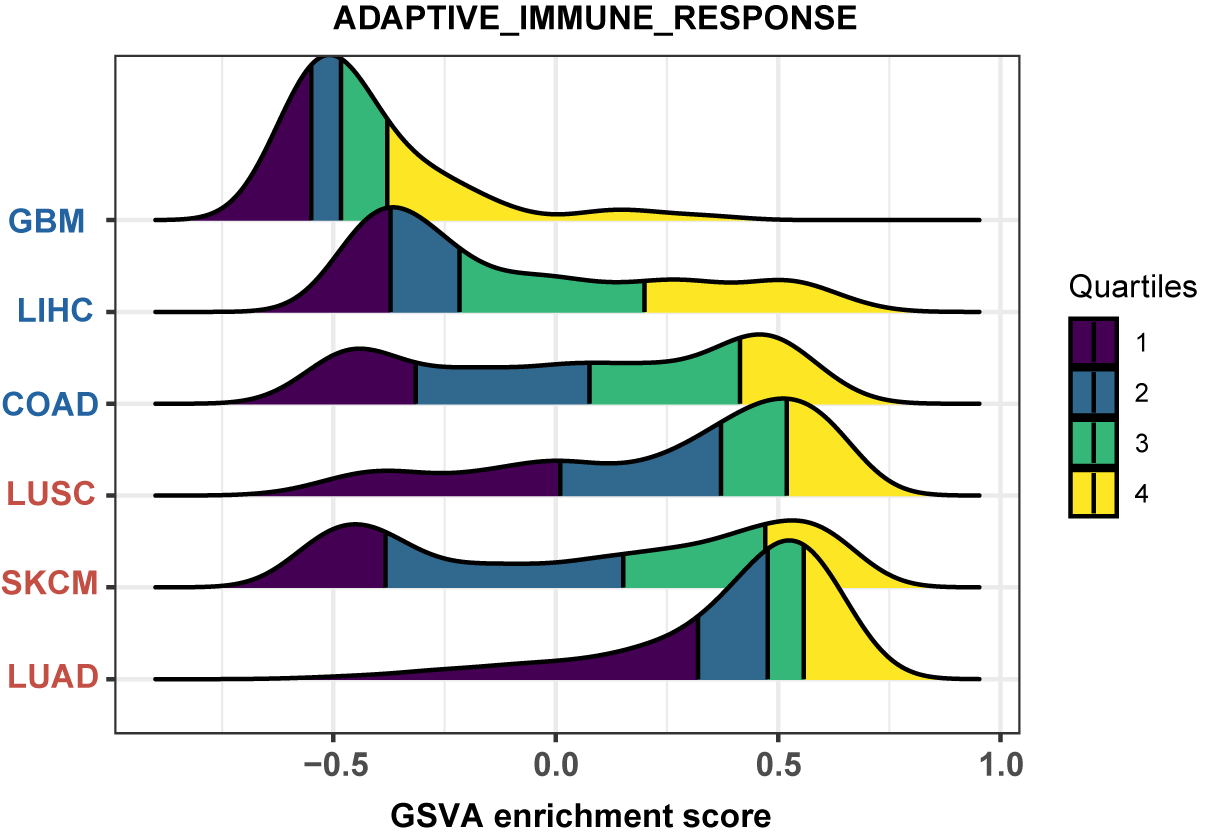

Supplement: Supplementary file 1 [file Image6.TIF]

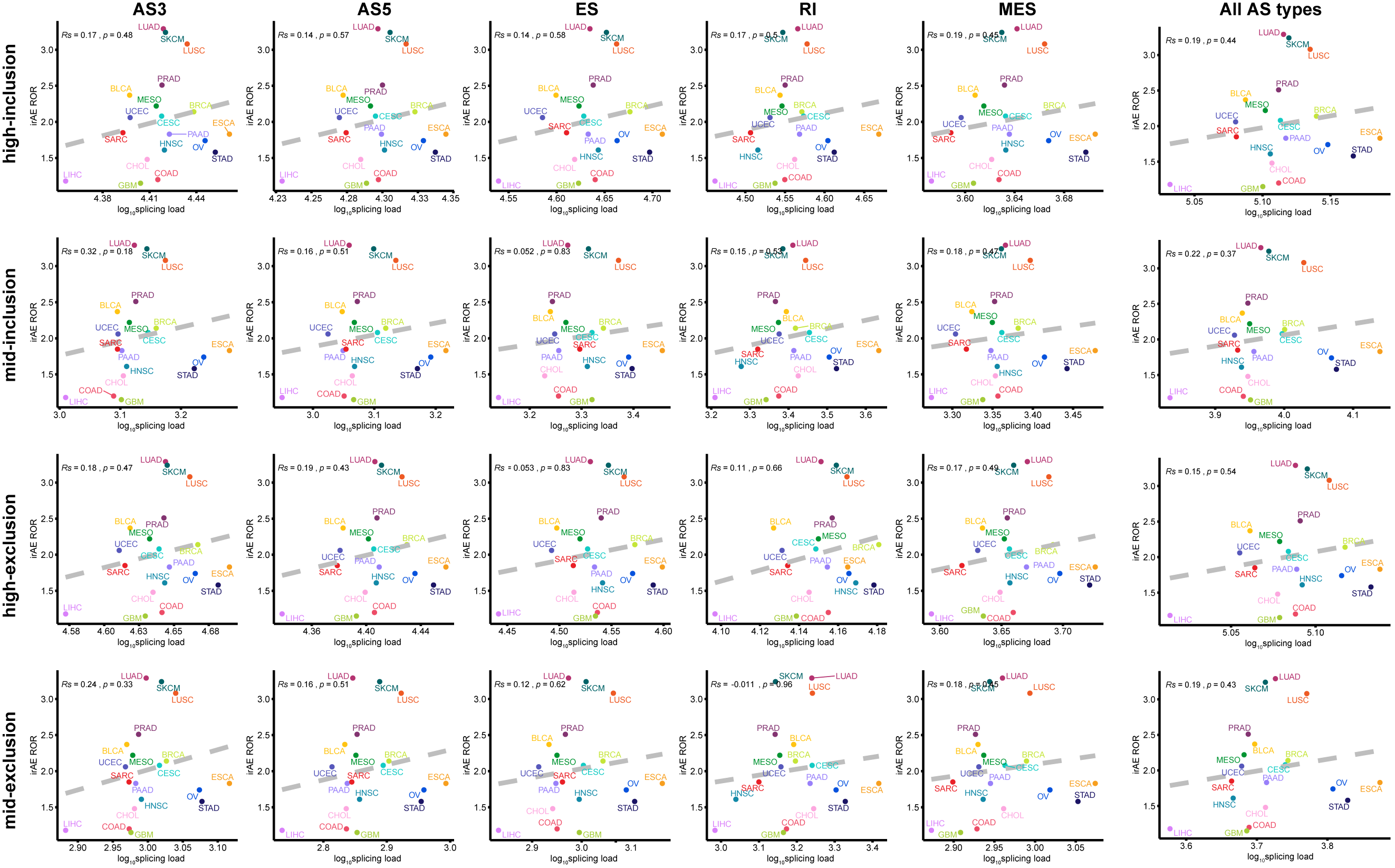

Supplement: Supplementary file 2 [file Image3.TIF]

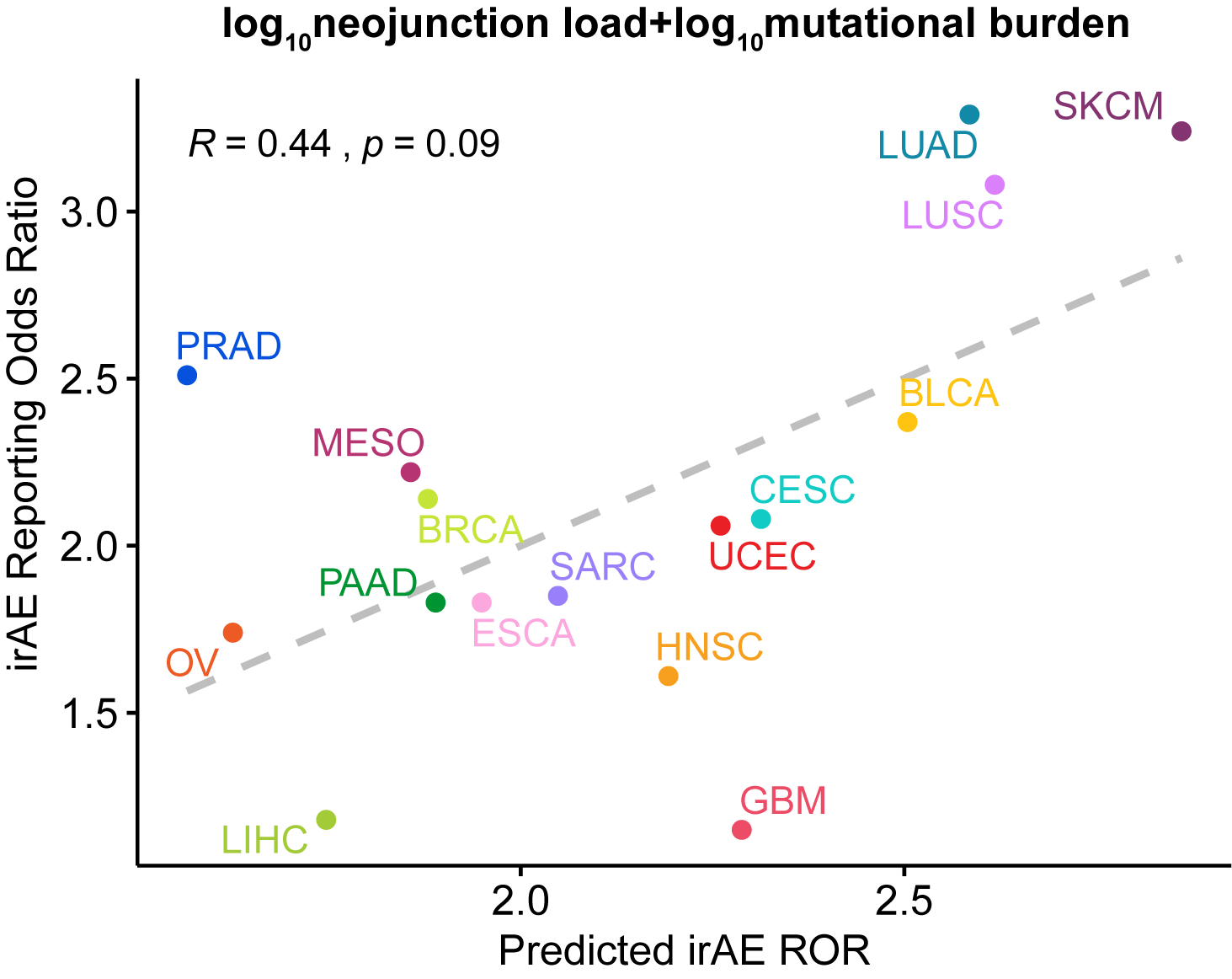

Supplement: Supplementary file 3 [file Image4.TIF]

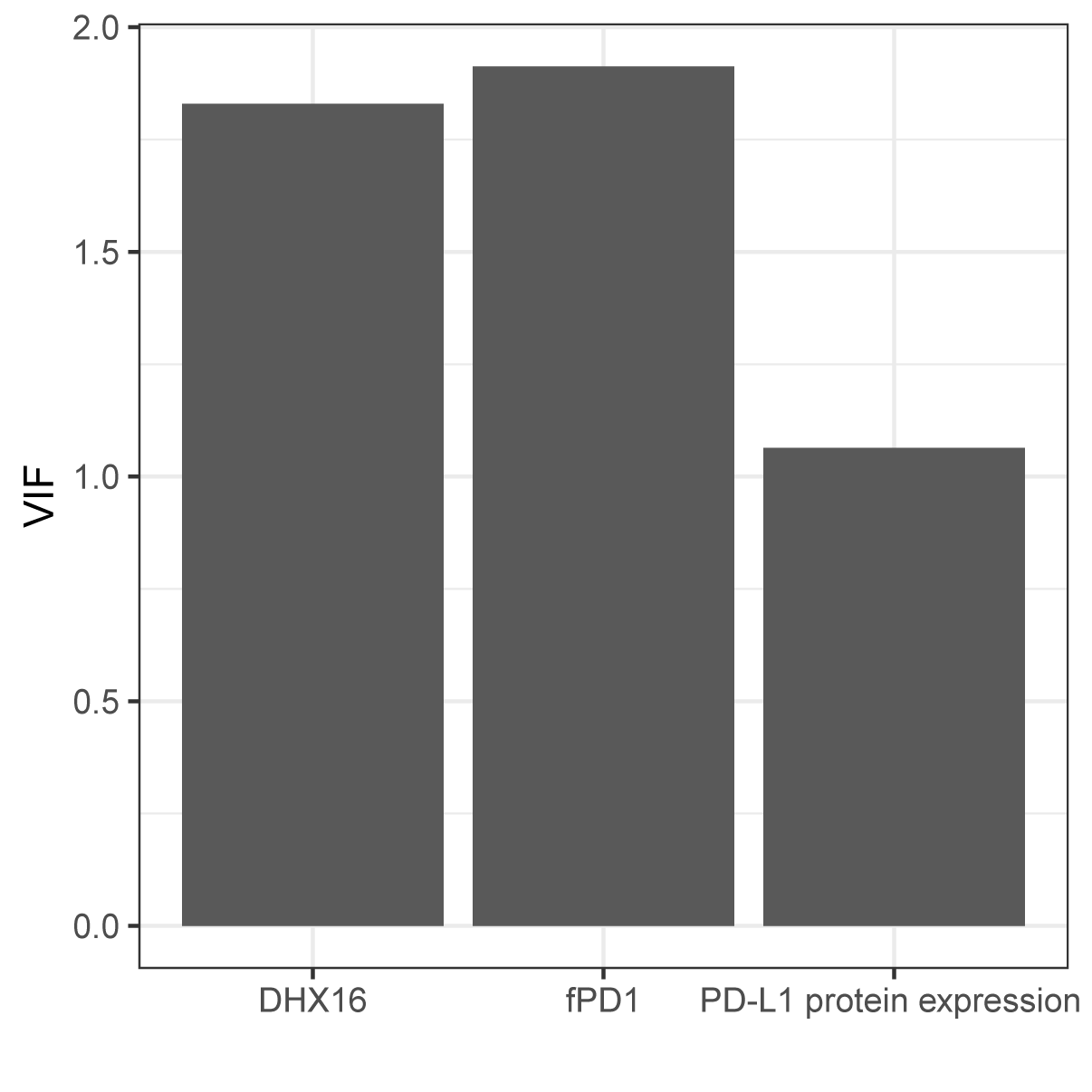

Supplement: Supplementary file 4 [file Image2.TIF]

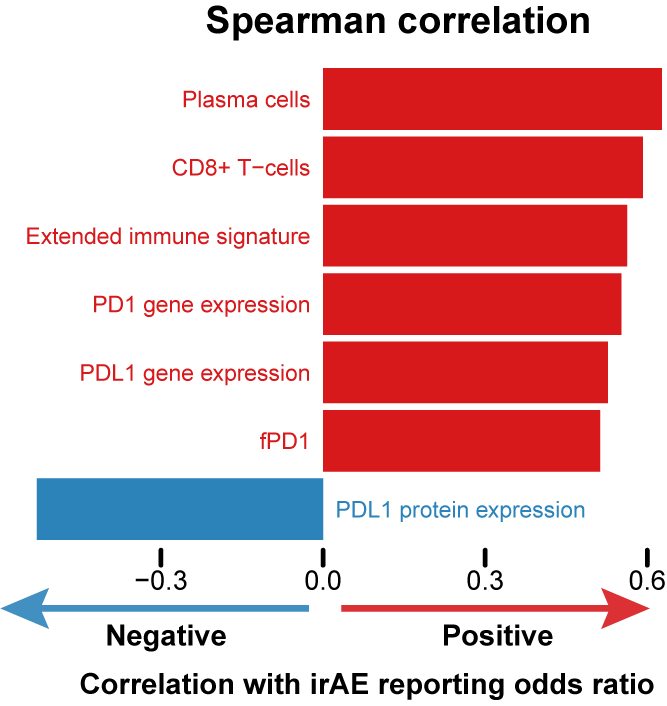

Supplement: Supplementary file 5 [file Image1.TIF]

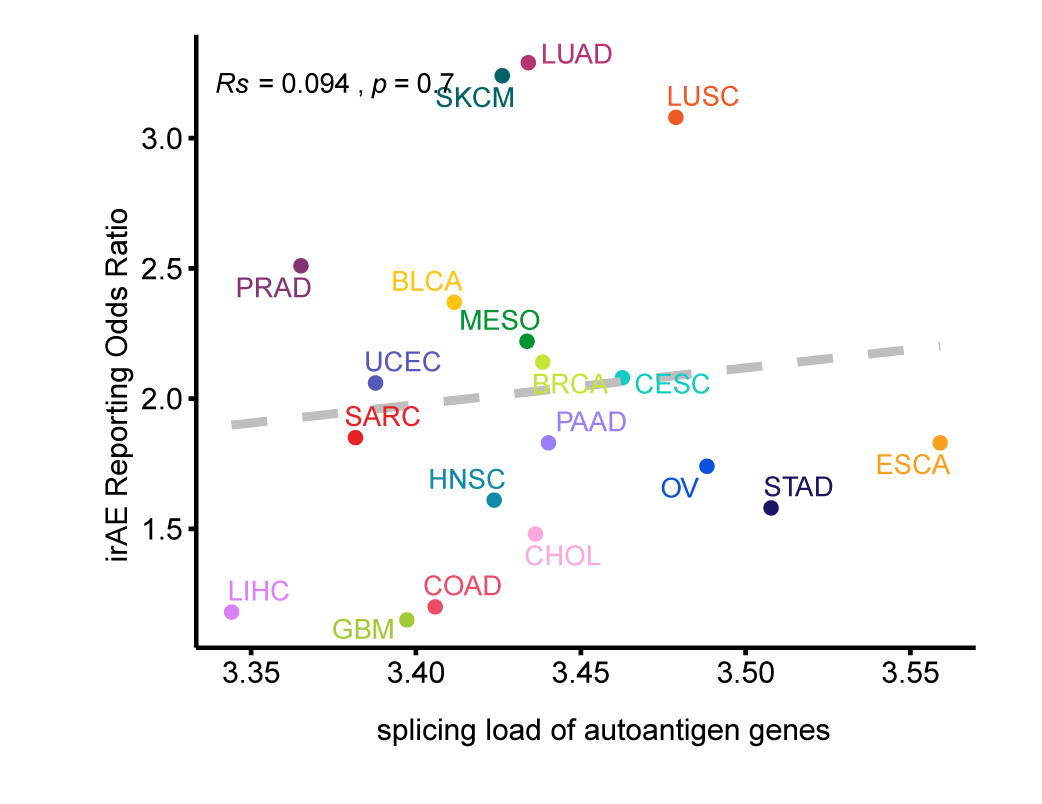

Supplement: Supplementary file 6 [file Image5.TIF]
